# A framework for clinical and translational research in the era of rigor and reproducibility

**DOI:** 10.1017/cts.2020.523

**Published:** 2020-08-19

**Authors:** Chris Wichman, Lynette M. Smith, Fang Yu

**Affiliations:** Department of Biostatistics, University of Nebraska Medical Center, Omaha, NE

**Keywords:** Rigor, reproducibility, team science, clinical translational research, replicability

## Abstract

**Introduction:**

Rigor and reproducibility are two important cornerstones of medical and scientific advancement. Clinical and translational research (CTR) contains four phases (T1–T4), involving the translation of basic research to humans, then to clinical settings, practice, and the population, with the ultimate goal of improving public health. Here we provide a framework for rigorous and reproducible CTR.

**Methods:**

In this paper we define CTR, provide general and phase-specific recommendations for improving quality and reproducibility of CTR with emphases on study design, data collection and management, analyses and reporting. We present and discuss aspects of rigor and reproducibility following published examples of CTR from the literature, including one example that shows the development path of different treatments that address anaplastic lymphoma kinase-positive (ALK+) non-small cell lung cancer (NSCLC).

**Results:**

It is particularly important to consider robust and unbiased experimental design and methodology for analysis and interpretation for clinical translation studies to ensure reproducibility before taking the next translational step. There are both commonality and differences along the clinical translation research phases in terms of research focuses and considerations regarding study design, implementation, and data analysis approaches.

**Conclusions:**

Sound scientific practices, starting with rigorous study design, transparency, and team efforts can greatly enhance CTR. Investigators from multidisciplinary teams should work along the spectrum of CTR phases, and identify optimal practices for study design, data collection, data analysis, and results reporting to allow timely advances in the relevant field of research.

## Introduction

Clinical and translational research (CTR) has been experiencing a resurgence since the mid-2000s along with an embracing of team science within the CTR community [1–4]. Around this same time, articles questioning or discussing the validity of published research results began to emerge in academic as well as nonacademic publications [5–7]. This perceived lack of trust in research has had an impact on investment and scalability [8] and has led to the formation of guidelines for how research studies should be reported, and a focus on scientific rigor and reproducibility by funding agencies, internal review boards, and editors alike [9,10].

Experience at our institution indicates that many investigators show interest in conducting CTR in their early research career. Our institution is currently funded with an Institutional Developmental Award Program Infrastructure for Clinical and Translational Research (IDeA-CTR). In the past 4 years, 68.4% (130 out of 190) of applicants for scholar program and pilot project awards were at assistant professor or lower rank. This high percentage of junior investigators applying for CTR funding indicates the need for education on rigor and reproducibility in CTR. Early career investigators and investigators new to the field of CTR alike, may have questions regarding the definition of the phases of CTR, how their research fits into the CTR spectrum, how to move their research from one phase to the next, and how to ensure rigor and reproducibility of their research.

While the definition of rigor is largely agreed upon, the definition of reproducibility is not [5,6,11]. Rigor means the study design, materials, conditions, data cleaning, analyses, interpretations, and reporting of results that are developed and documented in such a way as to produce unbiased results [12]. In contrast to rigor, reproducibility tends to have discipline-specific definitions ranging from an independent analyst getting the exact same result using the original data and code, to quantifying reproducibility with a measure such as the standard deviation of results [13–15]. As biostatisticians, we view reproducibility as the ability to obtain a consistent result when independent researchers utilize the same inclusion/exclusion criteria, study protocol, data cleaning rules, and analysis plan. Here consistency refers to parameter estimates being in the same direction and of similar magnitude with overlapping confidence intervals (CI). For example, if an original study estimated the effect of a 1 year increase in age on systolic blood pressure to be 2.3 (95% CI = (1.3, 3.3)) mmHg and the study repeated by an outside group under the same conditions obtained an estimated effect of a 1 year increase in age of 1.6 (95% CI = (0.4, 2.8)) mmHg on systolic blood pressure, the results of the two studies would be judged as consistent.

In this paper, we define CTR, and provide general and phase-specific recommendations for improving rigor and reproducibility of CTR with emphases on study design, data collection and management, analysis, and reporting. To guide the discussion and demonstrate the flow between translational research phases, we follow the development path of different treatments that address anaplastic lymphoma kinase-positive (ALK+) non-small cell lung cancer (NSCLC), as well as studies that demonstrate specific CTR challenges.

## Defining Clinical and Translational Research

One definition of CTR is moving research from bench to bedside to communities and back again. This definition seems clear enough, but categorizing any particular study into the CTR spectrum is challenging for new and established investigators alike. Broadly, T0 is defined as basic research, T1 as translating basic research to humans, T2 as translating findings to patients, T3 as translating research to general practice care, and T4 as translating research to populations or communities (Table 1). There is some disagreement where various study types should fall along the CTR spectrum. Fort et al describe the evolution of CTR definitions in the literature based on a clustering algorithm and gives a summary of the emerging consensus [16]. Surkis et al used a machine learning approach to classify studies based on a series of questions [17]. Main sources of disagreement for definition of studies along the CTR spectrum in the literature are whether Phase I clinical trials should be considered T1 or T2, if Phase IV clinical trials should be T2 or T3, if comparative effectiveness research should be T2, T3, or T4, and if health services research should be classified as T2 or T3. We created a compromise definition based on the goal of the research, defining all clinical trials as T2, and comparative effectiveness and health services research as T3, noting that there is disagreement about their classification (Table 1). Our suggestion is to classify a particular study into one of the CTR phases based on the goals of that study.

**Table 1.** Clinical and translational research classification definitions

	Goal	Examples
T0	Defining mechanisms of health or disease, animal or human	-Preclinical or animal studies
Basic research		-Association studies using large pre-existing datasets
		-Genome Wide Association Studies
T1	Applying understanding of mechanism to health of humans	-Preclinical development
Translation to humans		-Proof of concept
		-Biomarker study
		-Therapeutic targets identification
		-Drug discovery
T2	Developing evidence-based practice guidelines	-Phase I clinical trials*
Translation to patients		-Phase II clinical trials
		-Phase III clinical trials
		-Phase IV clinical trials*
T3	Comparing to widely accepted health practice	-Comparative effectiveness*
Translation to practice		-Pragmatic studies
		-Health services research*
		-Behavior modification
T4	Improving population or community health by optimizing interventions	-Population epidemiology
Translation to communities		-Policy or environmental change
		-Prevention studies
		-Cost effectiveness research
		-Patient preference/quality of Life

* Studies with disagreement in the literature as to their classification

**Fig. 1.**
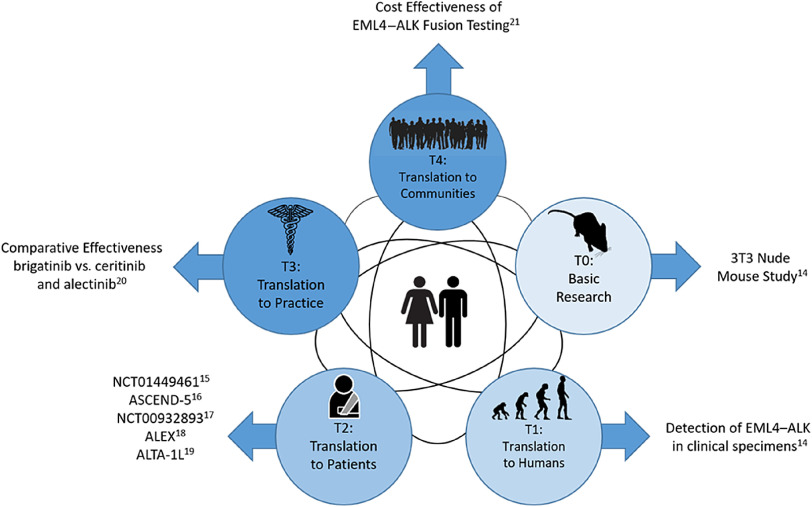
Phases of clinical translational research.

Figure 1 is an example of how small-molecule targeted cancer therapies are developed using ALK+ NSCLC as the target. Each circle represents a different phase of CTR and the black interconnecting lines indicate that the research path may be sequential, in parallel, or a hybrid of the two. The parallel aspect is demonstrated in Shaw et al which spans T0 and T1 [18]. The sequential aspect is demonstrated in the T2 phase with NCT01449461 [19], ASCEND-5 [20], NCT00932893 [21], ALEX [22], and ALTA-1L [23]. Finally, the T3 and T4 phases are represented by a comparative effectiveness [24] and a cost effectiveness study [25], respectively.

## Study Design

The first step in designing a study requires defining study objectives and hypotheses. Usually when moving research along the CTR spectrum there is an overarching objective, such as improving progression-free survival (PFS) in metastatic lung cancer patients. From this overarching objective, each phase of CTR will have “subobjectives” and testable hypotheses. As research moves along the CTR spectrum, each new study’s rationale is supported by results from the earlier phase studies or pilot studies. Hypotheses formed for CTR are built on the knowledge obtained from earlier phases (Table 2).

**Table 2.** Study design considerations

Topics	Description	T1	T2	T3	T4
Study objectives and scientific hypotheses	Idea or statement that provides a tentative explanation about certain facts or observations.	x	x	x	x
Sample Size	Required number of biological replicates to complete the study goals.	x	x	x	x
Power	Design parameter which specifies the probability of detecting a true intervention effect if one in fact exists. Typically power is set to be 80% or higher.	x	x	x	x
Stopping rule	Rules that are set in clinical trials to stop a study early for efficacy, futility or safety.		x	x	
Randomization	Process of assigning subjects to intervention groups based on chance alone. This minimizes differences across intervention groups and eliminates bias.	x	x	x	
Blinding	The subject, investigators, or both do not know the intervention assignment.	x	x	x	
Biologic Variables	Subject level variables that can play an important part in the disease process, such as age or gender.	x	x	x	x
Eligibility criteria	Definition of the population of interest. Generalization of the study results apply to this population.	x	x	x	x
Length of study	Duration of study.	x	x	x	x

The objectives and hypotheses should be matched with primary and secondary outcomes that are selected in advance. The study is designed around the primary question, and a clear question promotes good study design. To move to the next phase of the CTR spectrum, feasibility data or pilot information should be collected as secondary outcomes for planning purposes. In a T0/T1 research study, the primary objective is to build the knowledge base around the disease of interest, including basic science studies with animal models of human disease, or proof of concept studies. One example, from the T0/T1 phase, is a study by Soda et al which identifies novel transforming genes in NSCLC that can be used as therapeutic targets [18]. To meet their objective, researchers formulated a series of testable hypotheses using cell lines and mouse models to meet their goals. They identified a subset of NSCLC patients that express a transforming fusion kinase who have the EML4–ALK gene as a potential therapeutic target or a diagnostic molecular marker. In the continuation of these findings, a T2 research study was conducted to determine if crizotinib is superior to standard therapy in ALK-positive lung cancer (those that have the EML4–ALK gene) in an open label study, with the primary outcome of PFS [20].

Defining the study population is an important component of study design. The generalizability of the results relies on the eligibility criteria, which defines the population of interest. Early phase CTR (T1/T2) tend to have narrow eligibly criteria in order to reduce variability in the outcomes measured. This reduced variability is translated into differences that are more easily detected when testing hypotheses; however, these results are not widely generalizable. When moving further along the CTR spectrum (T3/T4), eligibility criteria are relaxed, thus allowing for more heterogeneity in the population. Subsequent results are more generalizable; however, with the increased variability, larger sample sizes are needed to detect the same or similar differences. In the T2 study comparing crizotinib to standard therapy, subjects were eligible if they had locally advanced or metastatic NSCLC that was positive for ALK rearrangements, with additional criteria regarding age and performance status [21]. Eligibility criteria should be clearly defined regardless as to whether they are strict or not. In order for a study to be reproducible (external validity), the population in which the original study was conducted must be known.

Regardless of where the research is on the CTR spectrum, good study design requires consideration of sample size and power of the study. Sample size justification in CTR serves a number of purposes. The primary purpose is to ensure there is adequate power to detect a clinically important difference specified by the scientific hypotheses. An underpowered study is nonreproducible. In an underpowered study, a statistically significant difference will appear by chance alone, which is not reproducible, or nonsignificant results with large *p*-values in an underpowered study may have been significant in a fully powered study. An important secondary purpose is in prespecifying a primary outcome variable. By prespecifying the primary outcome, this determines the analytical plan, and thus works to avoid reporting bias later in the study (the temptation of changing the primary outcome variable after the study has ended).

After the primary outcome is selected, the most important consideration for sample size is not statistical but scientific in nature, that of a clinically important difference (or effect) in the primary outcome. This difference would be meaningful for the scientific community and would be considered an important result. An estimate of a clinically important difference can come from expert opinion, scientific literature, and/or pilot data. An example of a clinically important difference can be found in the study comparing crizotinib vs. chemotherapy in ALK+ NSCLC [21]. Researchers determined that a 56% improvement in PFS, corresponding to a 2.5 month difference, with crizotinib (median PFS of 7.0 months) is a clinically important difference when compared to chemotherapy (median PFS of 4.5 months), requiring a sample size of 347. This is opposed to a statistically significant difference which is focused on obtaining a *p*-value less than 0.05. Any difference can be made to be statistically significant with a large enough sample size. If the crizotinib study had 10,000 subjects per group, they could detect a difference of 0.23 months between treatment arms, which would be considered a small nonimportant difference between groups.

Once the clinically important difference is defined, then type I error (alpha), power, and the study design (number of groups, type of study such as noninferiority or longitudinal) can be used to calculate a sample size for the study. Consideration should be given for variability, multiple comparison correction, and within subject correlation, as required.

**Fig. 2.**
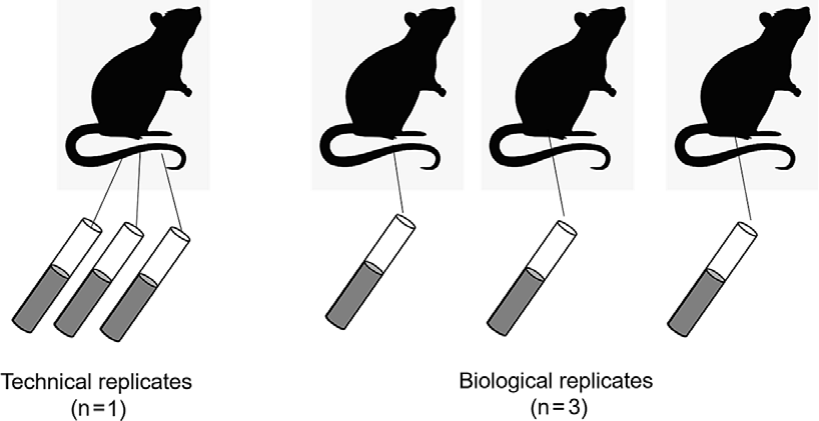
Comparison of Technical vs. Biological Replicates.

It is also important to consider the use of technical replicates vs. biological replicates, especially for T0 and T1 studies. Technical replicates are repeated measurements of the same sample, at roughly the same time, that measure variability of the process or experiment [26]. Biological replicates are measurements on independent biological samples that measure biologic variability [26]. Figure 2 shows an example of three technical vs. three biological replicates, notice how the technical replicates are all taken from the same mouse, whereas the biological replicates all come from separate mice (this is also applicable to human studies). Note that technical replicates cannot replace biological replicates in a study. Hypotheses are generally related to biological processes and variability at the biological level is needed for statistical comparisons. The use of technical replicates, in addition to biological replicates, will allow estimates of how reproducible the measurement equipment and protocols are for the experiment. Large technical variability can be attributed to numerous sources, including different lots of reagents, different equipment, or the samples were run on different days, or measurements were taken by different individuals. The reasons can be numerous and show the importance of good documentation of procedures. Once you have established that the technical variability is small, the analyses stage can be simplified by averaging over the technical replicates, giving one observation for each biological replicate for use in statistical models. The advantage of averaging over technical replicates is the analysis is greatly simplified, however a more complex analysis which includes the technical replicates in a mixed model allows us to better account for the nested structure of the data and multiple levels of variability.

During the T2 phase, particularly for clinical trials, sample size calculations and study designs often allow early stopping for efficacy, futility, or safety and should be approached in a rigorous manner. Early stopping at an interim analysis for safety or efficacy is necessary for ethical reasons, if one of the treatments is unsafe or superior to the other, it would not be ethical to continue to enroll subjects on either the unsafe or clearly inferior treatment. Additionally, stopping for futility is an important way to save patient and other resources for other more promising studies. In a randomized synbiotic trial to prevent sepsis in infants located in rural India, the study was stopped early for efficacy [27]. This study followed best practices for rigorous interim analyses, avoiding bias and maintaining operating characteristics of the study design by utilizing a Data Safety Monitoring Board so investigators remained blinded and multiple comparison adjustment determined by an *a priori* O’Brien–Fleming rule for stopping early.

Later phase studies in the CTR spectrum, T3 and T4, may utilize special designs, such as cluster randomization. As the name suggests, these studies randomize clusters, such as communities, schools, or hospitals. Some of the benefits of cluster randomized designs is that they can be logistically more feasible, they avoid within cluster contamination of the intervention groups, and they allow more people to be randomized in large public health trials. However, they require expertise in cluster sampling methods and analysis. These studies require that the correlation of individuals within a cluster be taken into account, inflating the sample size needs. Depending on the within cluster correlation, measured as the intraclass correlation, sample sizes may need to be inflated by a factor of 1.2 to 6.3 [28]. Regardless of the study design selected, care needs to be taken to design the study in a rigorous manner, utilizing best practices for the design chosen.

Randomization and blinding can apply to studies along the entire CTR spectrum in order to eliminate bias. At the T0/T1 phase, randomization and blinding can be applied to animal studies or doing assessments for biomarker studies. Animals should be randomly assigned to treatment groups, with sex as a stratification factor, meaning that female and male animals should be randomized separately. Randomization should be performed using a computer program or random digit table. Ideally, animals should have their own cages; this is because animals housed in the same cage are correlated with one another producing a “cage effect.” The cage effect is due to the animals interacting with each other and sharing food, water, and other resources. Researchers doing the assessments should be blinded, if possible, to the treatment assignment in order to produce an unbiased result.

Stratified randomization and block randomization can be used to minimize unbalance and to ensure that treatment groups are equally represented across strata and are balanced over time (blocking). Stratification is often used for smaller clinical trials in order to prevent imbalance in important prognostic factors at baseline. Study site is often used as a stratification factor in multicenter studies, along with gender, age, or disease stage as applicable. One limitation in using stratified randomization is that as the number of variables or factors increases the number of strata becomes large. In Shaw et al randomization was stratified by Eastern Cooperative Oncology Group performance status (0–1 vs. 2), presence of brain metastases (yes vs. no), and prior therapy with epidermal growth factor receptor kinase inhibitors (yes vs. no) [21], giving 6 strata. If researchers also wanted to stratify by age group (<65 vs. ≥65) and gender (male vs. female), then the number of strata goes up to 24. Depending on the total sample size, some of the strata could include a very small number of subject if any. Therefore, the number of stratification variables should be limited to those that are most important to keep the number of strata to a minimum.

Biologic variables, such as age and sex, should be considered both at the study design phase and at the analysis phase, as well as other important prognostic variables to prevent bias and obtain valid results. In T0/T1 studies, sex is an important biologic variable to consider for animal studies. Important differences by sex can be missed if only males or females are studied. Biologic variables should be planned for when calculating sample sizes and during the randomization process as stratification variables. If researchers want to be sure to capture treatment differences in each sex, the sample sizes are effectively doubled. If differences in treatment effects between sexes are not anticipated, then males and females can be combined for sample size calculation, but plans should include testing for sex differences in outcome. In later phase studies (T2–T4), biologic variables such as age, body mass index (BMI), race, gender, socioeconomic status, or underlying health conditions should also be considered at the design phase (eligibility criteria and stratification) and at the analysis phase in terms of reporting and model adjustment. One benefit of including stratification during randomization and analysis is that it can increase the power of the study by reducing variability in group comparisons [29].

Considerations made during the design phase of the study, including objectives, hypotheses, sample size considerations, randomization, stratification and biologic variables, as described above, should be laid out in a detailed protocol or manual of procedures to ensure scientific rigor and replicability/reproducibility. In early phase studies, this could be a document describing all the laboratory procedures that need to be followed, how records should be kept, and a log where protocol deviations can be listed. In clinical trials, a protocol is necessary. This document will describe the study in detail, giving the background, design, study schema, eligibility criteria, definitions of outcomes and adverse events, hypotheses, statistical considerations, and stopping criteria, among others. This document should be kept up to date, with any changes as amendments. Protocol deviations should be documented and reported, and the protocol should be available for review. For example, the protocol for the Shaw et al study of crizotinib in ALK-positive patients is available at NEJM.org [21]. The protocol corresponding to this clinical trial gives the background of the study science; gives the primary objective to demonstrate PF-02341066 (crizotinib) that is superior to standard of care in advanced NSCLC with an event involving the ALK gene locus; and multiple secondary objectives. It also provides the sample size estimation, statistical methods for addressing both primary and secondary objectives, procedures and adverse event reporting. This 96 page protocol document describes the study in much greater detail than the primary outcome paper could, allowing for other researchers to replicate this study in a separate patient population. It also allows for a critical assessment of their study methods and reporting, reviewers can determine whether the planned methods, analysis, and reporting match what is described in the primary outcome paper.

## Data Collection and Management

The research question, hypotheses, study design, and analysis plan will dictate the data to be collected for each subject. Typically, the amount and complexity of data collected increases as the translational research phase increases. Regardless of the amount or complexity of the data and/or translational phase, there are some tenets for good data collection:

1. each row represents a single observation;
2. each column represents a variable of interest;
3. each biological replicate should have a unique identifier;
4. each technical replicate should be tied to its parent biological replicate;
5. collect data to the highest degree of fidelity possible; categories can be created during analysis if needed;
6. each piece of information should be stored separately (e.g., follow-up date and status should be in separate columns);
7. maintain a data dictionary that spells out all definitions and abbreviations.

Common pitfalls in data collection are the mixing of data scales (e.g., recording temperature in degrees, Fahrenheit for some observation, and Celsius in others); inconsistent documentation for missing values; and analytical results being recorded using different criteria (e.g., pathology: one analyst records actual observed count; another analyst records categories of observed counts, such as <10,000). These pitfalls can be avoided or minimized by ensuring that all research staff who will be recording data are trained on the specific requirements as outlined in the research protocol and/or programming the database interface or spread sheet to only allow certain entries to be made. For example, in Excel®, one could use drop down menus to restrict entries, or in a database form, the interface can be programmed to accept only certain entries (e.g., using drop down menus or forcing the date to be recorded using a particular format). Or, if using a data base manager such as REDCap, data entry can be limited via data type restrictions embedded in the frontend worksheets when users populate in REDCap. One advantage of using a program such as REDCap is the audit trail created each time when data is entered or exported.

Note, if more than one person or site will be collecting data, the use of a spreadsheet is dangerous, since it is difficult to maintain an audit trail, data quality checks are not readily performed and the most recent version from each site/data entry person is extremely difficult to track.

In the basic research phase and small scale, single-center studies (T0, T1, and early T2), a simple spreadsheet is often sufficient to store the data. This is especially true if the data are going to be manually transcribed from a primary source (e.g., lab notebook or direct reading from an instrument) to the data collection instrument by a single person. For example, in Soda et al the nude mouse portion of the study would only require a simple spreadsheet with columns for mouse identifier (ID), group, and presence of tumor if the only goal were to determine which of the expression plasmids resulted in tumor formation [18]. However, because the study also incorporated an immunoblot analysis, some mechanism of tying the immunoblot with the appropriate mouse was needed. This could be as simple as using the mouse ID as part of the image file name. However, a more sophisticated approach would be to store the full file pathway for each image and subject in separate columns in a spreadsheet or in an image table within a database.

Some T0, T1, and T2 studies may benefit from more complex data storage strategies. Specifically, in genetic and -omics studies. These studies typically generate vast amounts of raw, transformed, and processed data. In addition, the meta-data encompassing demographic data, outcome data, processing dates, and processing software should be captured and stored. The data management issues that characterize these types of studies are beyond the scope of this article and interested readers are encouraged to review outside references [30].

In cases where multiple types or sources of data are required (e.g., demographic, clinical assessment, labs, etc. – typically mid-to-late T2, T3, and T4) a database approach is the best option to reduce errors and to store data efficiently. Databases store data in individual tables or forms based on the nature of the data. Each table or form must share, at a minimum, a unique subject ID so that data from different tables or forms can be “pulled” together for analysis. In addition, the use of electronic data capture (EDC) software may be useful. An EDC allows users to set up electronic forms, similar to hard copy worksheets. Whether directly inputting data into a database or utilizing front-end forms from an EDC, it is recommended to have a project data coordinator (PDC) on the research team. The PDC is responsible for constructing and maintaining the hard copy or EDC forms and the user interface for the database where research personnel enter the data as necessary.

For multicenter studies, the center (or study site) from which the observation originated must also be collected. In a spreadsheet, this is accomplished by adding a column for center and making the appropriate annotation for each observation. In a database, the center must be recorded on each table or form. With the observation or subject ID (and center ID – if required) in each table or form, a single data set representing the entire study can be constructed. For example, in the ASCEND-5 [21], ALEX [22], and ALTA-1L [23] studies, subjects were recruited from multiple centers and countries. Because the center is nested within its country, a separate data column for country is not necessarily needed.

In T4 studies, researchers are often utilizing national level or large aggregated databases. These datasets can be plagued with their own set of problems, such as: missing data; the collected data not being suitable for answering the research question posed; data coordinators and managers changing overtime and thus the organization of the data and the data collected over time may change; clinical or diagnostic definitions change over time; etc. From a data management perspective, both researchers and programmers must be aware of these limitations. One example of a database that changes over time is the United States Renal Data System which collects data on chronic kidney disease and end stage renal disease [31]. When purchasing access to this database, the most recent Researchers Guide along with the “What’s New” files (WNF) for each year from 2000 to the last completed year will be provided. The WNF are text files that delineate the changes made to the database structure, such as location of variables, new variables added, variables deleted for the year, and renaming of variables. Building a crosswalk between years and a table of data in common across years prior to querying the data is necessary to ensure as accurate a picture of the data as possible.

Researchers designing studies that utilize multiple forms and tables or receive data from multiple locations should consider employing a data team to include a project manager, data entry personnel, a data coordinator, a data monitor, and maybe an information technology (IT) specialist. The project manager is responsible for understanding the requirements of the protocol and ensuring all sites/researchers are adhering to the protocol and the appropriate data is being collected at the appropriate times. The data coordinator designs and maintains the forms and tables and ensures the appropriate versions are being utilized. Data entry personnel are trained on the protocol requirements and how to transcribe data that is not automatically populated into the database. The data monitor conducts data audits to ensure data quality (correctness and completeness). The data monitor also looks for potential data collection bottlenecks or issues with data collection and relays this to the project manager so that corrective action can be taken. Depending on the research teams’ hardware and software privileges, an IT specialist may also be necessary to navigate the intricacies of storing data electronically.

## Data Analysis

The rigor and reproducibility of CTR requires appropriateness of statistical methods for data analysis. The selection of the analytical methods for CTR of all phases should match the study intent, research design, and the type of data being collected for analyses. In this section, we will highlight several important aspects (Table 3) that should be considered when identifying statistical analysis methods.

**Table 3.** Data collection and analysis considerations.

Topics	Description	T1	T2	T3	T4
Data Collection and Management					
Data collection tool (subjective measurements)	Instruments used to collect data. These can take the form of surveys, interviews and focus groups, and observation.		x	x	x
Data management	Process by which data is acquired, stored, processed, and protected.	x	x	x	x
Data Quality	Refers to the state of the data, including completeness, cleanliness, and accuracy.	x	x	x	x
Data Analysis					
Statistical analytical method	Techniques to clean, transform, and model data to address research questions.	x	x	x	x
Model diagnostics	Statistical assumptions are checked to ensure they are met.	x	x	x	x
Intent to treat vs per protocol	Analysis technique defining the sample and analysis groups. Intent to treat analyzes all randomized subjects according to randomized groups providing unbiased estimates. Per protocol analyzes subsets of subjects according to the treatment received.		x	x	
Model validation	Verify validity of model(s) on independent data.	x	x	x	x
Missing data	Pieces of data that are not present for an observation, either because of nonresponse, attrition or because it wasn’t collected. The form of missingness can impact the validity of the study conclusions.	x	x	x	x

It is important to understand whether the study intent is exploratory or confirmatory. Exploratory research, often pilot studies, will be conducted when there is little theory or knowledge about the research questions. The goal of the exploratory research will be to generate hypotheses or refine existing hypotheses. The exploratory research may involve multiple outcomes and small sample sizes. In a Phase I/II trial, 137 ALK-rearranged NSCLC patients were recruited to assess the toxicity and efficacy of brigatinib [19]. Hence, the analyses are mostly descriptive and do not involve hypothesis testing. Contrarily, confirmatory research focuses on identifying reasons that explain the observed phenotypes or phenomenon and involve single or multiple hypothesis tests. When hypotheses involve comparisons among groups, different analytical methods will be used depending on whether the research focus is on equivalence, inferiority or superiority, or differences between groups.

The selection of analysis method also depends on whether the study is experimental or observational. Early phase CTR research, including T0/T1, T2, and some T3 research, may more easily apply experimental design to the study, given they have narrower eligibility criteria and may be conducted in a lab or other more controlled setting. Observational studies, on the other hand, are more likely to be used for later phase CTR, including comparative effectiveness studies in the T3 phase and policy impact assessment studies in the T4 phase. Observational studies tend to involve populations that are more general and assess the research questions in an empirical setting, in which it is not feasible to conduct a controlled experiment. In comparison to the controlled experiment, the observational study may not be able to collect some relevant information due to limited resources or lack of knowledge of when data was collected. Therefore, the covariates from groups under comparisons will not be balanced. Multiple regression or propensity score methodology will be useful to account for the imbalances among these covariates. For treatment studies when treatment assignment cannot be randomized due to ethnic reasons, causal inference techniques or propensity score matching or adjustment are particularly useful for analyses, as seen in the comparative effectiveness study to assess treatment effects of brigatinib vs. ceritinib and alectinib in crizotinib-refractory ALK+ NSCLC patients [20].

For all phases of CTR, the choice of the analytical method for studies will depend on the data under study, including the type and distribution of outcome variables, and inclusion of hierarchy or repeated measures. All analytical methods have their own assumptions and model diagnostics can be used to assess whether the chosen model is appropriate of an alternative that should be selected. Sensitivity analyses can be conducted to assess the change in the analytical results and inference when different models are applied.

Preparation is key, and investigators should be alert to issues related to study conduct and data collection when planning data analyses, including protocol violation, failure to recruit participants, and missing data. Protocol violations occur when some participants do not conform to the study protocol often in the context of in clinical trials from T2 and T3 CTR. Some examples of protocol violations are: failure to receive the assigned intervention, inappropriately receive another intervention under assessment, receive a prohibited concomitant intervention, or lack assessment of outcome due to loss of follow-up or other reasons [32]. Intent to treat analyses and per-protocol analyses have been developed to account for protocol violation issues. An intent to treat analysis should be considered the primary analysis and includes all participants randomized, according to randomized group, whereas a per-protocol analyses will include only those participants who complied with the study protocol. Intent to treat analyses will ensure unbiased estimation of intervention effect. Per-protocol analyses are most often conducted as part of a sensitivity analysis and can help assess the effects of intervention without influence of protocol violation or nonadherence. For example, in pragmatic clinical trials from T3 CTR, the participants are heterogeneous and may not adhere to the treatment protocol. The intent to treat analysis is recommended to maintain randomization and minimize the possible confounding when evaluating the intervention effects [33]. In addition, it is also important to assess how many participants are compliant to the study protocol and the exposure level of intervention for the participants in the intervention group. When participants of the trial received different levels of intervention, an evaluation of the treatment effects based on the actual exposure to intervention like dose-response or treatment on the treated will increase the reproducibility of the study, and provide better advice about the benefit of the evaluated intervention [34].

The data analyses also need to account for the possibility of unexpectedly high failure in participant recruitment or excessive withdrawals during study conduct. Failure in participant recruitment or excessive withdrawals results in smaller sample size, which could lead to underpowered study. To overcome this underpower challenge, several steps in the analysis can be made to address this. One possibility is that the analyses can be adjusted to use continuous outcomes vs. categorical outcomes, when appropriate. Another is when a comparative study involving multiple groups is under investigation, groups with similar influence in magnitude or directions can be combined to maximize the group size, decrease the number of tests, and optimize the study power. Additionally, exact tests may be preferred over asymptotic tests in small sample situations.

Early drop-out leads to an important missing data issue that adds complexity to the data analyses, especially for later phase CTR. In studies with a large number of subjects, missing data will frequently occur due to various reasons. For example, participants may have nonresponse for questions related to income, administration of medication, or other sensitive questions. In another scenario, the participants may become too fatigued to complete the assessment, or have severe side effects that prevent them to continuing in the study. Analytical methods for handling missing data depend on the type of missing data mechanism that governs the missingness: 1) missing at random when the propensity of missing is not related to observed data or missing data, 2) missing completely at random when the propensity of missing is related to observed data, but not related to missing data, and 3) missing not at random. A popularly used method for handling missing data is complete case analysis, which excludes subjects with missing data. The complete case analysis is especially useful when the study involves a small proportion of missing data. Other commonly considered methods include maximum likelihood, multiple imputation, and full Bayesian methods [35,36].

When the goal of the study is prediction, statistical model validation is crucial for assessing the accuracy of the identified statistical models for predicting outcomes [37]. Model validation can be conducted internally or externally depending on the availability of external data. In internal validation, the data can be split into a training dataset for developing the prediction model and a validation dataset to validate the prediction performance of the preidentified model. External validation is also very important, though it is not always possible if external data is unavailable. The use of external data that is similar to the testing data will help assess the reproducibility of the prediction model; however, if the external data is quite different to the testing data, the external validation will be useful for assessing the model generalizability.

Development of a statistical analysis plan prior to initiation of the CTR study will help investigators avoid HARKing (hypothesizing after the results are known), and will improve reproducibility and transparency of the study [38]. HARKing can occur under different scenarios. For example, the investigator may change their *a priori* hypotheses to different hypotheses with significant results in order to improve their chance of publication. The original study design may fail to collect and/or adjust for important biologic variables, or could conduct many subgroup analyses, or try different choices of cut-points based on data in hand to categorize continuous data, and only report analyses associated with significant results. These practices may lead to irreproducibility issues due to their vulnerability to the small sample sizes or high dependence on individual data. The statistical analysis plan includes key components, such as study objectives, hypotheses to be tested, outcomes and variables that will be collected during the study, and the statistical methods, which contains enough detail to allow other researchers to independently replicate the results.

Developing an analysis plan before data collection will facilitate peer review and maintain continuity of the research team to ensure appropriateness of the analytical method in addressing the research question. The predeveloped analysis plan also helps to prevent confirmation bias (deciding how to handle outliers or missing data, or in meta-analyses which study to include or exclude based on whether the results were in the direction expected or desired by researcher). The predeveloped analysis plan will also allow the researchers to differentiate theory-driven hypotheses instead of data-driven hypotheses. Any adjustment to the statistical analysis plan after data has been collected should be justified and the results from those new analyses should be used cautiously as they were developed post hoc, and may be driven by the collected data.

## Results Reporting

A rigorous report of the study can facilitate the study reproducibility and increase study impact. To ensure the rigor of the study report, sufficient detail about the study objectives, design, methods, and materials should be included. When discrepancies occurred between the original study design and the study conduct, it is important to include justification and detailed discussion regarding those discrepancies. Examples include reporting protocol violations, changes in procedures over time, and changes in planned sample size vs. actual sample size. The study report should also include an accurate report of study results and make appropriate study inferences and conclusions without extrapolating the study findings.

A number of guidelines have been developed for different types of CTR studies to improve reporting completeness, transparency, and scientific rigor. The clinical and translational researchers can follow those guidelines based on the CTR study type, or adapt these protocols when reporting their studies. Specifically, the investigators can refer to the Consolidated Standards of Reporting Trials (CONSORT) [10] for clinical trials, the Strengthen the Reporting of Observational Study in Epidemiology (STROBE) [39], the Preferred Reporting Items for Systematic Reviews and Meta-Analyses (PRISMA) [40], the Standards for Reporting of Diagnostic Accuracy Studies (STARD) [41], and the Transparent Reporting of a Multivariable Prediction Model for Individual Prognosis of Diagnosis (TRIPOD) [42]. In addition, Prager et al (2019) defined general reporting criteria for the use of academic publishing [43].

Many CTR studies start as pilot studies with institution support to assess the study feasibility and obtain preliminary data in support of grant preparation and developing future large-scale studies [44]. Regardless of whether the studies yield positive or negative results, the results from such pilot studies can provide valuable information for refining study processes and hypotheses and guiding future research with respect to design, instruments, and methods. Therefore, the investigators should be encouraged to be transparent regarding their study purpose and publish their study findings even when the study results are negative.

## Discussion

It is important to utilize a rigorous and reproducible approach to advance CTR along the spectrum. In this paper, we focused on how to conduct high quality CTR, and provided general and phase-specific guidelines regarding study design, data collection and management, data analyses, and result reporting. There are many challenges in progressing research along CTR spectrum; and by utilizing best practices in scientific methods, these challenges can be minimized.

Utilizing a team science approach, clinical and translational researchers should involve investigators and translational collaborators from different disciplines from the study’s initiation, and integrate the interdisciplinary expertise and knowledge for forming study concepts, design, and methodology. For example, a team with basic scientists, clinicians, and public health experts will aid in moving the research along CTR spectrum, leading from T0/T1 through T4. It is also important to involve community partners to incorporate their experience in practice, especially for patient-centered outcome research, and facilitate the dissemination of the findings to communities.

There is existing work conducted by Lapchak et al on rigor and reproducibility of stroke translational research. Lapchak focused more on T1 laboratory animal studies to T2 human trials for drug development. They discussed rigor applied to study design. In this paper, we emphasized rigor and reproducibility of clinical translation research under different areas. We considered CTR of all phases, and provided recommendations related to not only study design but also data management and analyses.

To meet the goal of rigorous, reproducible/replicable science, there is a need for transparency [45]. Transparency allows for clear understanding of design, methods, and analysis. Without transparency, science may be rigorous, but will not be reproducible or replicable. One way to encourage transparency is through the open science initiative [46]. Through this initiative, researchers are encouraged to preregister studies and to share data and analysis code. With this information available, the scientific community can compare the planned study to the final product. The researcher will be extra careful in all steps of the study because they know the scientific community will have access to the prestudy plan, the actual data, and the analysis code. By preregistering studies or protocols, researchers will be less inclined to “fish” for significant results (also known as p-hacking), hoping for some positive result to publish. Often researchers do not want to make data publicly available with hopes of publishing more themselves. Besides transparency, another argument for publishing deidentified data is that researchers attempting to replicate your study, either through a formal replication process [47] or as part of a meta-analysis, will lead to many more citations of the original work, increasing its visibility.

To avoid publication bias, all study results, whether positive or negative, should be published. The scientific community is recognizing the importance of publishing studies with negative results, if only to avoid duplication of research efforts and possibly show more promising directions of research. There are now journals devoted to publishing negative results, such as a *PLoS ONE* collection, called *Missing Pieces* [48], which presents inconclusive, negative findings, or failed replications. The *Journal of Negative Results in BioMedicine* [49] ceased publication in 2017 because many journals followed their lead in publishing negative studies that they no longer saw a need for this specialized journal.

The majority of research efforts have been made on the early phase CTR. Surkis et al [17] assessed PubMed ID (PMIDS) of all publications indexed in PubMed to past or present Clinical Translational Science Award (CTSA) grant number for five participating CTSA institutions, and randomly selected 40 papers per institution. Two institutions were invited to manually classify these 200 studies into phases along the CTR spectrum using agreed criteria. Out of 185 papers with clear classification, 106(57.3%) papers belonged to T0 basic science category, while 18(9.7%) papers were classified as T1/2 CTR, and 44(23.8%) were classified as T3/T4 CTR. This evidence implied that there may be a good proportion of T0 research that fail to advance to later phase CTR, or more resources should be allocated to promote CTR research advancement.

The advancement of CTR is not necessarily sequential across spectrums. As shown in Figure 1, the scientific learning from different parts of the spectrum of CTR can feed into each other at any level and promote the CTR research of lower and higher stages. For example, the recent breakout of COVID-19 infection has motivated CTR of different phases to be undertaken in parallel to understand the mechanism of the virus, identify strategies for infection prevention, and treatment of COVID-19. A T0 CTR by Lu et al (2020) studied the phylogenetic sequencing of the coronavirus to understand the similarity and difference between COVID-19 and other coronaviruses, like MERS and SARS, as well as the outbreaks’ origins [50]. Simultaneously, a multi-institutional phase 2 trial is underway [51] to assess the efficacy of Remdsivir in treating COVID-19 infections. Remdsivir, developed by Gilead Sciences, was selected due to its effects in treating other coronaviruses in animal models. Wu et al (2020) did a T4 epidemiologic study to estimate the domestic and global public health risks of coronavirus infection epidemics. Their study indicated the importance of developing a large scale public health COVID-19 intervention to avoid independent self-sustaining outbreaks in major cities globally [52].

In summary, sound scientific practices, starting with rigorous study design, transparency, and team efforts can greatly enhance CTR. Investigators new to CTR should familiarize themselves with best practices for study design, data collection, data analysis, and results reporting to allow timely advances in their field of research. Research teams that incorporate investigators along the spectrum of CTR phases, biostatisticians, and, depending on phase, community partners can lead to successful CTR research.

## References

[1] Wadman M . US translational-science centre gets under way. Nature 2012; 481(7380): 128. 22237088 10.1038/481128a

[2] Wuchty S , Jones BF , Uzzi B . The increasing dominance of teams in production of knowledge. Science 2007; 316(5827): 1036–1039. 17431139 10.1126/science.1136099

[3] Bennett LM , Gadlin H . Collaboration and team science: from theory to practice. Journal of Investigative Medicine 2012; 60(5): 768–775. 22525233 10.231/JIM.0b013e318250871d PMC3652225

[4] NIH. Institutional Development Award (IDeA) Program Infrastructure for Clinical and Translational Research (IDEA-CTR)(U54). In:2017.

[5] Baker M . 1,500 scientists lift the lid on reproducibility. Nature 2016; 533(7604): 452–454. 27225100 10.1038/533452a

[6] Collins FS , Tabak LA . Policy: NIH plans to enhance reproducibility. Nature 2014; 505(7485): 612–613. 24482835 10.1038/505612a PMC4058759

[7] Yong E . Psychology’s Replication Crisis is Running Out of Excuses. *The Atlantic* 2018.

[8] Osherovich L . Hedging against academic risk. Science-Business eXchange 2011; 4(15): 1–2.

[9] Sciences NAo. Reproducibility and Replicability in Science. Washington, DC: National Academies Press, 2019. 31596559

[10] Schulz KF , et al. CONSORT 2010 Statement: updated guidelines for reporting parallel group randomized trials. Open Medicine 2010; 4(1): e60–e68. 21686296 PMC3116666

[11] Goodman SN , Fanelli D , Ioannidis JP . What does research reproducibility mean? Science Translational Medicine 2016; 8(341): 341ps312. 10.1126/scitranslmed.aaf5027 27252173

[12] Health NIo. Enhancing Reproducibility through Rigor and Transparency [Internet]. (https://grants.nih.gov/policy/reproducibility/index/htm)

[13] Shabanali R . Basic Concepts: Reproduction vs Replication - The Secret Behind Every Evolving System [Internet]. (webmindset.net/reproduction-vs-replication-the-secret-behind-every-evolving-systen)

[14] IUPAC. Compendium of Chemical Terminology, 2nd edition (the “Gold Book”). Oxford: Blackwell Scientific Publications; 1997.

[15] Patil P , Peng R , Leek J . A statistical definition for reproducibility and replicability. BioRxiv 2016. 10.1101/066803.

[16] Fort DG , et al. Mapping the evolving definitions of translational research. Journal of Clinical and Translational Science 2017; 1(1): 60–66. 28480056 10.1017/cts.2016.10 PMC5408839

[17] Surkis A , et al. Classifying publications from the clinical and translational science award program along the translational research spectrum: a machine learning approach. Journal of Translational Medicine 2016; 14(1): 235. 27492440 10.1186/s12967-016-0992-8 PMC4974725

[18] Soda M , et al. Identification of the transforming EML4-ALK fusion gene in non-small-cell lung cancer. Nature 2007; 448(7153): 561–566. 17625570 10.1038/nature05945

[19] Gettinger SN , et al. Activity and safety of brigatinib in ALK-rearranged non-small-cell lung cancer and other malignancies: a single-arm, open-label, phase 1/2 trial. The Lancet Oncology 2016; 17(12): 1683–1696. 27836716 10.1016/S1470-2045(16)30392-8

[20] Shaw AT , et al. Ceritinib versus chemotherapy in patients with ALK-rearranged non-small-cell lung cancer previously given chemotherapy and crizotinib (ASCEND-5): a randomised, controlled, open-label, phase 3 trial. The Lancet Oncology 2017; 18(7): 874–886. 28602779 10.1016/S1470-2045(17)30339-X

[21] Shaw AT , et al. Crizotinib versus chemotherapy in advanced ALK-positive lung cancer. The New England Journal of Medicine 2013; 368(25): 2385–2394. 23724913 10.1056/NEJMoa1214886

[22] Peters S , et al. Alectinib versus Crizotinib in Untreated ALK-Positive Non-Small-Cell Lung Cancer. The New England Journal of Medicine 2017; 377(9): 829–838. 28586279 10.1056/NEJMoa1704795

[23] Camidge DR , et al. Brigatinib versus Crizotinib in ALK-Positive Non-Small-Cell Lung Cancer. The New England Journal of Medicine 2018; 379(21): 2027–2039. 30280657 10.1056/NEJMoa1810171

[24] Reckamp K , et al. Comparative efficacy of brigatinib versus ceritinib and alectinib in patients with crizotinib-refractory anaplastic lymphoma kinase-positive non-small cell lung cancer. Current Medical Research and Opinion 2019; 35(4): 569–576. 30286627 10.1080/03007995.2018.1520696

[25] Djalalov S , et al. Cost effectiveness of EML4-ALK fusion testing and first-line crizotinib treatment for patients with advanced ALK-positive non-small-cell lung cancer. Journal of Clinical Oncology 2014; 32(10): 1012–1019. 24567430 10.1200/JCO.2013.53.1186

[26] Blainey P , Krzywinski M , Altman N . Points of significance: replication. Nature Methods 2014; 11(9): 879–880. 25317452 10.1038/nmeth.3091

[27] Panigrahi P , et al. A randomized synbiotic trial to prevent sepsis among infants in rural India. Nature 2017; 548(7668): 407–412. 28813414 10.1038/nature23480

[28] Parker DR , Evangelou E , Eaton CB . Intraclass correlation coefficients for cluster randomized trials in primary care: the cholesterol education and research trial (CEART). Contemporary Clinical Trials 2005; 26(2): 260–267. 15837446 10.1016/j.cct.2005.01.002

[29] Friedman LM , et al. Fundamentals of Clinical Trials. New York: Springer; 2010.

[30] Jones MB , et al. The new bioinformatics: integrating ecological data from the gene to the biosphere. Annual Review of Ecology, Evolution, and Systematics 2006; 37: 519–544.

[31] System USRD. 2018 USRDS annual data report: Epidemiology of kidney disease in the United States. 2018.

[32] Gupta SK . Intention-to-treat concept: A review. Perspectives in Clinical Research 2011; 2(3): 109–112. 21897887 10.4103/2229-3485.83221 PMC3159210

[33] Sedgwick P . Explanatory trials versus pragmatic trials. BMJ 2014; 349: g6694. 25395503 10.1136/bmj.g6694

[34] Yu F , Hein NA , Bagenda DS . Preventing HIV and HSV-2 through knowledge and attitudes: a replication study of a multi-component community-based intervention in Zimbabwe. PLoS One 2020; 15(1): e0226237. 31914165 10.1371/journal.pone.0226237 PMC6949002

[35] Dempster AP , Laird NM , Rubin DB . Maximum likelihood from incomplete data via the EM algorithm. Journal of the Royal Statistical Society Series B (Methodological) 1977; 39(1): 1–38.

[36] Ibrahim JG , Chu H , Chen MH . Missing data in clinical studies: issues and methods. Journal of Clinical Oncology 2012; 30(26): 3297–3303. 22649133 10.1200/JCO.2011.38.7589 PMC3948388

[37] Steyerberg EW , Harrell FE, Jr. Prediction models need appropriate internal, internal-external, and external validation. Journal of Clinical Epidemiology 2016; 69: 245–247. 25981519 10.1016/j.jclinepi.2015.04.005 PMC5578404

[38] Kerr NL . HARKing: hypothesizing after the results are known. Personality and Social Psychology Review 1998; 2(3): 196–217. 15647155 10.1207/s15327957pspr0203_4

[39] Von Elm E , et al. The Strengthening the Reporting of Observational Studies in Epidemiology (STROBE) statement: guidelines for reporting observational studies. Annals of Internal Medicine 2007; 147(8): 573–577. 17938396 10.7326/0003-4819-147-8-200710160-00010

[40] Moher D , et al. PRISMA statement. Epidemiology 2011; 22(1): 128. 21150360 10.1097/EDE.0b013e3181fe7825

[41] Bossuyt PM , et al. The STARD statement for reporting studies of diagnostic accuracy: explanation and elaboration. Annals of Internal Medicine 2003; 138(1): W1–W12. 12513067 10.7326/0003-4819-138-1-200301070-00012-w1

[42] Collins GS , et al. Transparent reporting of a multivariable prediction model for individual prognosis or diagnosis (TRIPOD): the TRIPOD statement. The TRIPOD Group. Circulation 2015; 131(2): 211–219. 25561516 10.1161/CIRCULATIONAHA.114.014508 PMC4297220

[43] Prager EM , et al. Improving transparency and scientific rigor in academic publishing. Brain and Behavior 2019; 9(1): e01141. 30506879 10.1002/brb3.1141 PMC6346653

[44] Moore CG , et al. Recommendations for planning pilot studies in clinical and translational research. Clinical and Translational Science 2011; 4(5): 332–337. 22029804 10.1111/j.1752-8062.2011.00347.x PMC3203750

[45] NIH. Rigor and reproducibility [Internet] [cited Feb 2, 2020]. (https://www.nih.gov/research-training/rigor-reproducibility)

[46] Munafo M . Open science and research reproducibility. Ecancermedicalscience 2016; 10: ed56. 27350794 10.3332/ecancer.2016.ed56 PMC4898932

[47] Brown AN , Cameron DB , Wood BD . Quality evidence for policymaking: I’ll believe it when I see the replication. Journal of Development Effectiveness 2014; 6(3): 215–235.

[48] PLOS. The Missing Pieces: A Collection of Negative, Null and Inconclusive Results [Internet], 2015. (https://collections.plos.org/missing-pieces)

[49] *Journal of Negative Results in BioMedicine* [Internet], 2002. (https://jnrbm.biomedcentral.com/) 10.1186/1477-5751-1-2 PMC149424 12459050

[50] Lu R , et al. Genomic characterisation and epidemiology of 2019 novel coronavirus: implications for virus origins and receptor binding. The Lancet 2020; 395(10224): 565–574. 10.1016/S0140-6736(20)30251-8 PMC7159086 32007145

[51] NIH. NIH clinical trial of remdesivir to treat COVID-19 begins [Internet], 2020. (https://www.nih.gov/news-events/news-releases/nih-clinical-trial-remdesivir-treat-covid-19-begins)

[52] Wu JT , Leung K , Leung GM . Nowcasting and forecasting the potential domestic and international spread of the 2019-nCoV outbreak originating in Wuhan, China: a modelling study. The Lancet 2020; 395(10225): 689–697. 10.1016/S0140-6736(20)30260-9 PMC7159271 32014114

